# Mercury accumulation in vegetable *Houttuynia cordata* Thunb. from two different geological areas in southwest China and implications for human consumption

**DOI:** 10.1038/s41598-020-80183-7

**Published:** 2021-01-08

**Authors:** Qingfeng Wang, Zhonggen Li, Xinbin Feng, Ao Wang, Xinyu Li, Dan Wang, Leilei Fan

**Affiliations:** 1 Department of Resources and Environment, Zunyi Normal College, Zunyi, 563006 People’s Republic of China; 2 State Key Laboratory of Environmental Geochemistry, Institute of Geochemistry, Chinese Academy of Sciences, Guiyang, 550081 People’s Republic of China; 3 University of Chinese Academy of Sciences, Beijing, 100049 People’s Republic of China; 4 Zunyi Product Quality Inspection and Testing Institution, Zunyi, 563006 People’s Republic of China

**Keywords:** Risk factors, Environmental impact

## Abstract

*Houttuynia cordata* Thunb*.* (*HCT*) is a common vegetable native to southwest China, and grown for consumption. The results suggested that THg contents in all parts and MeHg in underground parts of *HCT* in Hg mining areas were much higher than those in non-Hg mining areas. The highest THg and MeHg content of *HCT* were found in the roots, followed by the other tissues in the sequence: roots > leaves > rhizomes > aboveground stems (THg), and roots > rhizomes > aboveground stems > leaves (MeHg). The average THg bioaccumulation factor (BCF) of *HCT* root in the Hg mining area and in non-Hg mining areas could reach 1.02 ± 0.71 and 0.99 ± 0.71 respectively, indicating that *HCT* is a Hg accumulator. And the THg and MeHg contents in all tissues of *HCT*, including the leaves, were significantly correlated with THg and MeHg content in the soil. Additionally, preferred dietary habits of *HCT* consumption could directly affect the Hg exposure risk. Consuming the aboveground parts (CAP) of *HCT* potentially poses a high THg exposure risk and consuming the underground parts (CUP) may lead to a relatively high MeHg exposure risk. Only consuming the rhizomes (OCR) of the underground parts could significantly reduce the exposure risk of THg and to some extent of MeHg. In summary, *HCT* should not be cultivated near the Hg contaminated sites, such as Hg tailings, as it is associated with a greater risk of Hg exposure and high root Hg levels, and the roots should be removed before consumption to reduce the Hg risk.

## Introduction

Mercury (Hg) is a highly toxic, mobile, and chemically stable element ^1^. It presents health concerns because of the risk of exposure to humans through various pathways, including inhaling contaminated air, consuming contaminated food and drinking water, and direct skin contact^2^. Guizhou province, situated in the center of the Circum-Pacific mercuriferous belt, is a globally important center of Hg production. Long-term mining activities in this region have caused serious Hg pollution ^3^, and also increased the exposure risks in the local population through the presence of a high Hg concentration in various environmental media, especially in food stuff for human consumption, including meat ^4^, crops (especially rice) ^4,5^, and vegetables ^6,7^.

*Houttuynia cordata* Thunb. (Saururaceae; *HCT*), which is native to Asian countries, has heart-shaped leaves and stoloniferous rhizomes (Fig. S1 in the Supporting Information), and prefers warm, moist, and shady environments. As an extremely popular vegetable in southwest China, its consumption history could be date back to the Eastern Han dynasty (25–220 A.D.)^8^. It is also used among diverse cultures across Asia for medicinal purposes ^9^. According to an earlier report^10^, Guiyang (the capital city of Guizhou province) has the largest consumption amount of *HCT* in China, with an average daily consumption amount over 15 tons (average daily per capita consumption at least 30.74 g/day/person). Another report suggested that the average daily per capita consumption was even up to 400 g/day/person in Guiyang City ^11^. Our investigation also showed the per capita intake of *HCT* by adult in Kaiyang county of Guiyang city was as high as 76 g/day/person^12^. Moreover, previous studies have confirmed the ability of *HCT* to strongly uptake lead ^13^, arsenic ^14^, and cadmium ^15,16^. It is therefore a good candidate species for soil remediation at sites contaminated by heavy metals. It also exhibits a high Hg uptake. Qian, X. et al. ^17^ investigated 259 wild plants belonging to 49 genera in 29 families growing on wasteland comprising tailings in Wanshan Hg mining area to screen for possible phytoremediation species, and *HCT* was the only one has relatively high Hg accumulation ability that potently consumed in large quantities by the local population. Previous studies have reported many other Hg accumulation plant^18–22^, such as *Pteris vittate*^19^, *Sesbania drummondii*^20^ and *Jatropha curcas*^23^, while almost all such plants are not edible for human beings. Moreover, recently reports have paid close attention to health risks of associated with the consumption of local food items in contaminated environments^6,12,24–29^. A research of our group indicated *HCT* has the highest Hg concentration and bioaccumulation factor (BCF) value among dozens of vegetables, fruits and crops in Hg mining areas ^30^. And the highest total mercury (THg) content of *HCT* was approximately 100 times higher than the upper limit for vegetables (10 µg/kg, fresh weight; FW) in China’s General Standard for Contaminants in Foods ^31^. Our previous study recognized that consuming *HCT* was an important human Hg exposure route in Kaiyang mercury mine in central Guizhou and *HCT* has the highest mercury content by compare with other five vegetables^12^. Therefore, relatively high accumulation of Hg and large consumption amount of *HCT* may pose a significant risk of Hg exposure to local population.

However, previously studies didn’t pay much attention on the Hg distribution among different tissues of *HCT* and their associated health risk implications, since the eaten part of *HCT* varied considerable among regions in China, and different dietary habits may lead to significant regional differences in Hg exposure. In Guizhou and Yunnan province, people prefer to consume the underground parts (roots and rhizomes) of *HCT*, in particular the tender rhizomes, whereas the aboveground parts (aboveground stems and leaves) are generally preferred in Sichuan province and Chongqing city (see Fig. S1 for a schematic diagram of *HCT* plant parts). Moreover, it has been suggested that the Hg content (dry weight basis, DW) in the aboveground parts is higher than in the underground parts ^17^, contrasting with the common acceptance that plant roots contained a higher Hg content than other plant parts ^22,32,33^. And there is still insufficient information about Hg concentrations in *HCT* growing in different areas, especially in locations with lower Hg contamination levels.

In present study, samples of *HCT* and rhizosphere soil were collected for analysis from an Hg mining area (Danzhai Hg mine, DZ) and a non-Hg mining area (Zhijin county, ZJ), both located in Guizhou province. Our objectives were to: (1) determine the Hg accumulation and distribution of different *HCT* tissues between the two locations; (2) analyze the effect of soil Hg content on *HCT* Hg content in different tissues; and (3) evaluate the Hg exposure risks presented by varying dietary habits related to *HCT*. Our overall aim was to provide insights into the potential risks associated with *HCT* consumption.

## Material and methods

### Study areas

**Figure 1:**
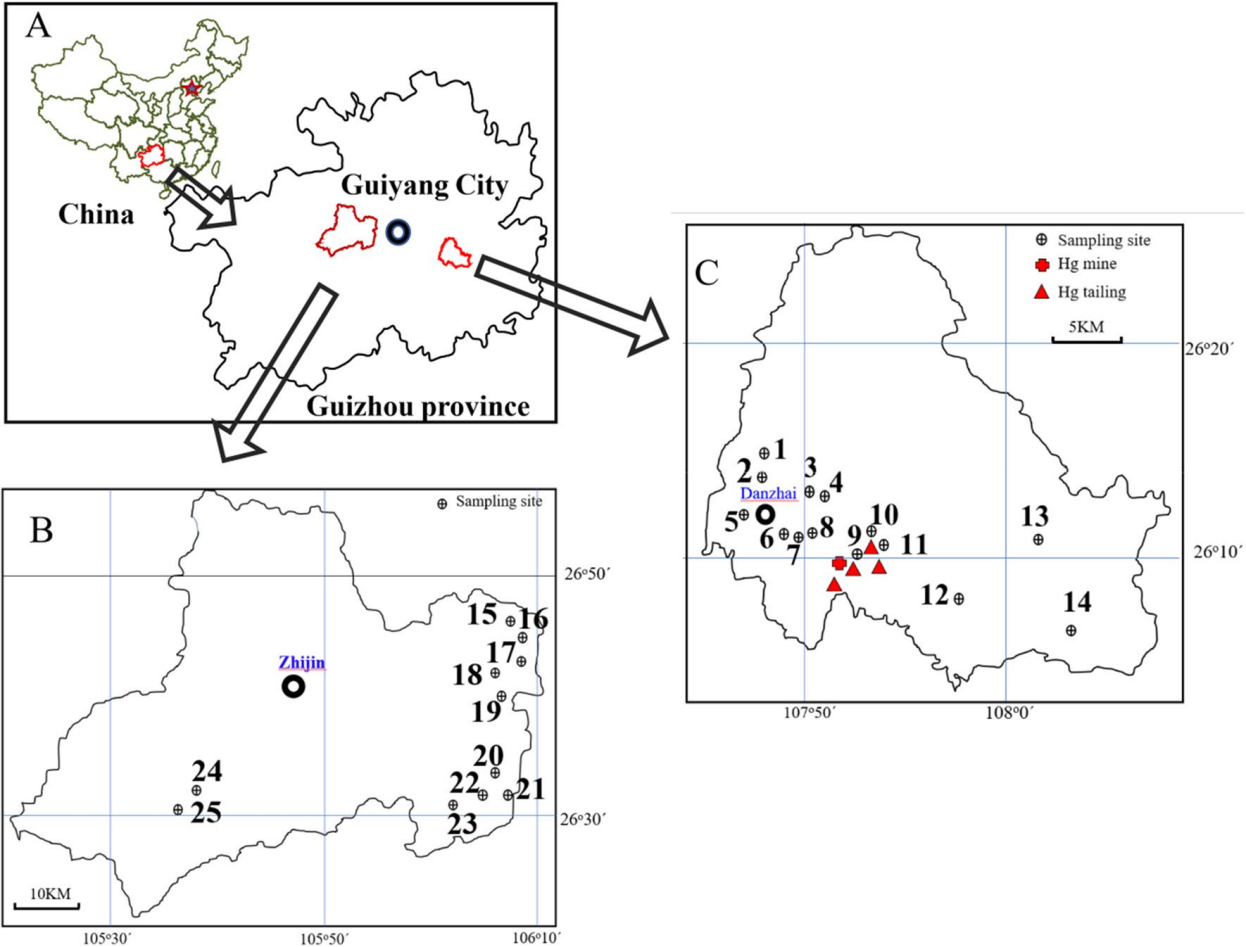
Sampling locations and historical mercury (Hg) mining sites in the study areas.

The study areas were in the center-west and southeastern areas of Guizhou province, located in ZJ (area, 2868 km^2^; population, 1.231 million [2018]; altitude, 860–2262 m a.s.l.) and Danzhai (DZ; area, 940 km^2^; population, 0.174 million [2017]; altitude, 700–1100 m a.s.l.) counties, respectively (Fig. 1). The two counties are approximately 260 km apart; both have a well-developed karst landform and subtropical monsoon climate (average temperature, approximately 12.6–17.2 °C). The local climate and environmental conditions are very suitable for *HCT* growth.

Danzhai Hg mine, where mining activity dates back to the 1950s, is located in the south of DZ (Fig. 1C). Its ores also contain Au and As, and it is sometimes referred to as Danzhai Au-Hg mining area^34^. The mine closed in the 1990s. A rough estimate is that 186 million tons of waste were produced and deposited close to the mining site without treatment, causing severe pollution of the local environment ^35^. Zhijin (Fig. 1B) is one of the main anthracite coal-producing areas of Guizhou province, and forms part of the famous Zhijin-Nayong coalfield. The coal Hg content is relatively lower (< 0.1 mg/kg) than in other coal-producing regions in Guizhou ^36^. And according to the Geochemical Atlas of China ^37^, ZJ is located in an area of a relatively lower Hg background in Guizhou province.

### Sample collection

Local populations forage for *HTC* in the wild and also cultivate it as a vegetable crop. Therefore, *HCT* samples were both collected from vegetable gardens and from sites where it grows wild (Fig. 1). The sampling sites in DZ were mainly distributed around Danzhai city and near the Hg tailings, while those in ZJ county were mainly distributed in rural areas. Samples of *HCT* and rhizosphere soil (approximately 0.5 kg and 1 kg, respectively, from each site) were collected between June and September, 2019. In total, 14 *HCT* and 14 rhizosphere soil samples were collected in DZ, and 11 *HCT* and 11 rhizosphere soil sample were collected in ZJ, respectively, placed in polyethylene ziplock bags, and stored in a cooler at 4 °C for transportation. They were taken back to the laboratory, where the *HCT* samples were cleaned and separated into roots, rhizomes (underground stems), aboveground stems, and leaves (Figs. S1 and S2), according to previous studies and the standard for food stuff determination^6,23,38,39^.The weights of different parts of each sample were recorded and then dried at 50 °C for approximately 5 days. The rhizosphere soil samples were also dried at 50 °C. All solid samples were then ground and passed through a 0.150-mm nylon sieve.

### Analytical methods and quality assurance

#### THg and MeHg in *HCT* and rhizosphere soil samples

The THg concentrations in the *HCT* and rhizosphere soil samples were analyzed using a Milestone Direct Mercury Analyzer with AMA 254 software (Model DMA-80) according to Environmental Protection Agency (EPA) method 7473 ^40^. The basic steps of THg measurement consist in placing a known amount of milled solid sample in a nickel or quartz sample holder. The sample holder is then introduced in a quartz furnace, where it is heated up to 200 °C for 60 s for sample drying and 650 °C for 105 s for Hg reduction and volatilization. Air is used as combustion and carrier gas. Combustion gases containing released Hg^0^ are then flushed through a cobalt-manganese oxide catalyst, where interferents like halogen compounds, nitrogen oxides and sulfur oxides are retained. Hg^0^ is selectively trapped in a gold-coated sand amalgamator and then released from it by heating at 850 °C for 3 s. And then the released Hg^0^ is carried to an atomic absorption detection cell, where the absorbance from the radiation emitted by a mercury lamp is measured at 253.7 nm^41,42^. MeHg concentrations in the soil and *HTC* tissue samples were measured using gas chromatography–cold vapor atomic fluorescence spectrometry (GC–CVAFS)(GC: Model TRACE 1300E, Thermo Fisher Scientific, USA; CVAFS: Model 2500, Tekran, Canada) after potassium hydroxide (KOH)-methanol/solvent extraction, ethylation, and purge-and-trap collection, according to the method used in a previous study ^40,43^.

#### Calculation of bioaccumulation factor (BCF) and chronic daily intake (CDI) value

The bioaccumulation factor (BCF) is an index of the ability of organisms to accumulate a particular metal with respect to its concentration in their environment ^17,44^, which in the case of our experiment is the soil substrate. The BCF values for THg and MeHg in different *HCT* tissues can be calculated according to Eq. (1).

1 $${\text{BCF = }}\frac{{{\text{C}}_{{{\text{HCT}}}} }}{{{\text{C}}_{{{\text{soil}}}} }}$$

where ${\text{C}}_{{{\text{HCT}}}}$ is the concentration in different *HCT* tissues (DW, μg/kg), and ${\text{C}}_{{{\text{soil}}}}$ is the soil Hg content (μg/kg).

Hg exposure risk to *HCT* was evaluated by using chronic daily intake (CDI) values (Eq. (2)) for the general adult population, as recommended by US EPA ^45–47^.

2 $${\text{CDI }} = \, \left( {{\text{C }} \times {\text{ IR }} \times {\text{ EF }} \times {\text{ ED}}} \right)/\left( {{\text{BW }} \times {\text{ AT}}} \right)$$

where C is the THg and MeHg concentration in *HCT* (unit in μg/kg, fresh weight basis (FW)); IR is the ingestion rate (kg/person/day); EF is the exposure frequency (365 days/year); ED is the exposure duration (76.3 years, equivalent to the average life span); BW is the average body weight (55.9 kg); and AT is the average time of exposure for noncarcinogens (AT = 365 × ED). The value of average life span is 76.3 years in China^7^. The average body weight (55.9 kg) in the references of a previous study^6^.

#### Quality assurance and quality control

Quality control measures were in reference of several previous studies^17,48^, including method blanks, triplicates, and several certified reference materials. The limits of detection were 0.01 μg kg^−1^ for THg and 0.002 μg kg^−1^ for MeHg respectively. The certified reference materials (CRM) of GBW10020 (Orange foliage, THg: 150 ± 13 ng/g) and TORT-2 (Lobster Hepatopancreas, MeHg: 152 ± 13 ng/g) in this study were used for THg and MeHg analysis and the average recoveries were 106.3% and 91.7%. The recoveries on matrix spikes (MeHgCl solution) of MeHg for *HCT* and soil digest were in the range 91—121%. And the certified reference GBW07405 (Yellow–red soli, THg: 290 ± 40 ng/g) and ERMCC580 (Estuarine sediment, MeHg: 75.5 ± 3.7 ng/g) in this study were used for soil THg and MeHg analysis, and the average recoveries were 104.7% and 101.6%. The mean THg concentration of CRM GBW10020 was determined at 146 ± 11 ng g^−1^ (N = 6) and of GBW07405 at 297 ± 53 ng g^−1^ (N = 6), whereas the mean MeHg concentration of CRM TORT-2 was 154 ± 21 ng g^−1^ (N = 6) and of CRM ERMCC580 was 74.3 ± 4.1 ng g^−1^ (N = 6), which were all comparable well with its certified values. The relative standard deviation (RSD) of duplicate analysis for Hg concentration data in this study all less than 8%. Statistical analysis was performed with SPSS 21.0 software, and the figures were created using Origin 9.0.

## Results and discussion

### THg and MeHg in the underground and aboveground parts of *HCT*

**Figure 2:**
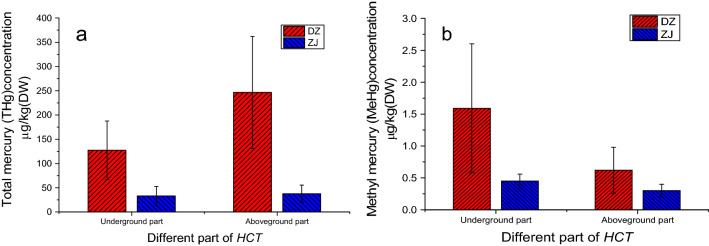
Total mercury (THg) and methylmercury (MeHg) concentrations in different tissues of *Houttuynia cordata* Thunb. (*HCT*) (dry weight [DW]) in Danzhai (DZ) and Zhijin (ZJ).

Varying dietary habits between regions mean that different parts of *HCT* are often preferred: underground parts, including roots and rhizomes, or aboveground parts, including aboveground stems and leaves. The results of the statistical analysis of Hg concentrations in the under- and aboveground parts for both DZ and ZJ are shown in Table 1 and Fig. 2 (based on DW). The THg and MeHg concentrations in both *HCT* parts among different sampling sites are shown in Fig. S3. The results clearly showed that the THg and MeHg contents in *HCT* varied widely among different collection sites. By comparing with these two areas, significant difference (*p* < *0.05*) was found for the THg content in both under- and aboveground parts and MeHg in the underground parts between DZ and ZJ, with the Hg mining area are higher than the non-Hg mining area. And no significant difference in MeHg for the aboveground parts between these two areas. For different tissues of a plant, THg content of the aboveground parts was significantly higher than that of underground parts both in DZ and ZJ. While, MeHg content of the aboveground parts was significantly lower than the aboveground parts in DZ and no significant difference was found of MeHg content between the under- and aboveground parts in ZJ. It should also be noted that *HCT* samples collected near Hg tailings (#9–11; Fig. 1) contained a significantly higher content of THg and MeHg compared with other sample sites in DZ, and this indicated that the presence of Hg tailings can greatly increase *HCT* Hg content in the surrounding farmland.

**Table 1:** Total mercury (THg) and methylmercury (MeHg) concentration in the underground and aboveground parts of *Houttuynia cordata* Thunb. (*HCT*).

Items	Study areas	Underground part, μg/kg				Aboveground part, μg/kg			
		Range (DW)	AM ± SD (DW)	Range (FW)	AM ± SD (FW)	Range (DW)	AM ± SD (DW)	Range (FW)	AM ± SD (FW)
THg	DZ	71–272	127 ± 61	15–56	26 ± 13	138–541	246 ± 116	21–83	38 ± 18
	ZJ	19–83	33 ± 20	4–17	7 ± 4	25–52	36 ± 7	4–8	7 ± 1
MeHg	DZ	0.45–3.49	1.59 ± 1.01	0.09–0.72	0.33 ± 0.21	0.30–1.39	0.62 ± 0.36	0.05–0.21	0.10 ± 0.06
	ZJ	0.33- 0.68	0.45 ± 0.11	0.05–0.11	0.07 ± 0.02	0.16–0.53	0.30 ± 0.10	0.02–0.08	0.05 ± 0.02

*AM* Arithmetic mean, *SD* standard deviation. *DW* dry weight basis, *FW* fresh weight basis.

THg content in the aboveground parts of *HCT* collected from DZ was approximately twice than that of the underground parts (DW), but only slightly higher in comparison to material collected from ZJ. In DZ, the THg and MeHg contents (measured in DW) could be as high as 272 μg/kg and 3.49 μg/kg in the aboveground parts, and 541 μg/kg and 1.39 μg/kg in the underground parts, respectively. The average values for THg and MeHg content in the underground parts in DZ (127 ± 61and 1.59 ± 1.01 μg/kg, DW, respectively), were about four and three times as high as in ZJ, respectively (33 ± 20 and 0.45 ± 0.11 μg/kg, DW). Furthermore, THg and MeHg in the aboveground parts in DZ (246 ± 116 and 0.62 ± 0.36 μg/kg, DW, respectively) were approximately six and two times as high as in ZJ (36 ± 7 and 0.30 ± 0.10 μg/kg, DW, respectively). THg concentrations in all samples (N = 14) collected from DZ exceeded the limit for vegetables specified in the national guidance (10 μg/kg, FW), while only 18.2% (N = 11) of the underground parts in ZJ exceeded this limit (Fig. 1). Moreover, our results showed that the highest MeHg content (1.59 ± 1.01 μg/kg) in the underground parts collected from DZ was higher than in other vegetables (0.023–2.5 μg/kg), meat (0.26–0.85 μg/kg), poultry (0.56–2.4 μg/kg)^4^, and corn (0.20 ± 0.34 μg/kg)^38^ collected from Hg mining areas in Guizhou. The lowest MeHg content (0.30 ± 0.10 μg/kg) in the aboveground parts of *HCT* collected from ZJ was only slightly higher than in corn (0.20 ± 0.34 μg/kg)^38^, which is considered to be a low MeHg staple cereal. This indicates that the Hg exposure risk vary both with different dietary habits and whether the *HCT* source area is in an Hg or non-Hg mining area.

It was commonly accepted that Hg is taken up/accumulate by the roots for most plants and less is translocated towards the shoot^22,32,33,49^. Though some studies^50,51^ showed a high translocation of Hg towards the aerial parts, most of Hg was found in the root of a plant. In contrast with previous studies, THg concentration in the aboveground parts of *HCT* was significant higher that in the underground parts in both Hg (DZ) and non-Hg mine areas (ZJ). And this result also consisted with several previous studies shown in Table 2 that THg content (DW) is always higher in the above- compared with underground parts. THg content in the aboveground parts of *HCT* collected from DZ and ZJ is much lower than in severely Hg- contaminated areas, such as mine tailing wastelands in Wanshan Hg mining area^17^, but is much higher than in samples collected from farmland in Sichuan and Chongqing^52^ and a coal mining area in China^53^. THg content in the underground parts of *HCT* in DZ is comparable with the results of our previous study in Kaiyang Hg mining area, Guizhou^12^. This result once again confirmed that *HCT* is a plant with high translocation of Hg from root to the aerial parts just like *Jatropha curcas*^50^ and *Cyrtomium macrophyllum*^51^. And for MeHg, previous studies showed that its high solubility in lipids and easily been taken up by plants^5,54^. Our results showed that MeHg content of the underground parts was significantly higher than that of the aboveground parts in DZ and no significant difference was found between the under- and aboveground parts in ZJ. This also indicates that *HCT* has relatively high translocation ability of MeHg. In summary, although the aboveground parts of *HCT* demonstrated a less contamination than the underground parts in terms of MeHg, consumption of the former (mainly in Sichuan and Chongqing) could pose a greater risk of THg exposure, whereas the consumption of roots and rhizomes may pose a greater risk of exposure to MeHg.

**Table 2:** Concentrations of total mercury (THg, mg/kg) in *Houttuynia cordata* Thunb. (*HCT*) reported in the literature.

Resource of *HCT*	Underground parts mg/kg, DW	Aboveground parts mg/kg, DW	References
Danzhai, Guizhou Zhijin, Guizhou	0.127 ± 0.061 0.039 ± 0.020	0.246 ± 0.115 0.036 ± 0.007	This study
Kiayang Guizhou	0.081 ± 0.121	–	Our previous study Wang et al.^12^
Farmlands, Sichuan and Chongqing	0.02–0.03	0.03–0.05	Chen et al.^52^
Wanshan mercury mining area, Guizhou	1.5 ± 0.81	1.8 ± 0.72	Qian et al.^17^
A coal mining area, China	0.009	0.003	Li et al.^53^

### Hg accumulation in *HCT* tissues

**Figure 3:**
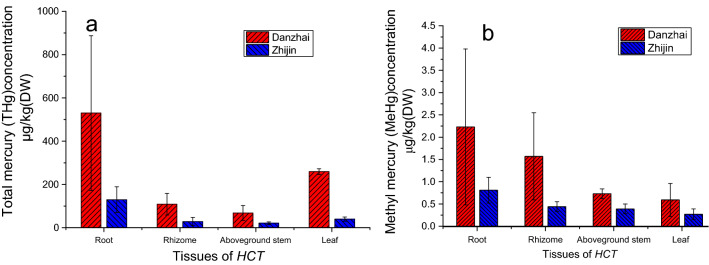
The bioaccumulation factor (BCF) value in different tissues of *Houttuynia cordata* Thunb. (*HCT*) in Danzhai (DZ) and Zhijin (ZJ). The error bar indicates one standard deviation.

Table 3 and Fig. 3 showed the results of THg and MeHg content in *HCT* tissues. Hg content in different tissues and soils collected from the study area is shown in Figs. S4 and S5. The results clearly showed that THg and MeHg content in all four types of *HCT* tissues collected from DZ is much higher than that from ZJ. Mean THg concentrations in *HCT* tissues were ranked from high to low as follows: roots > leaves > rhizomes > aboveground stems. Mean MeHg concentrations in *HCT* showed a different order, again from high to low: roots > rhizomes > aboveground stems > leaves. The results were in agree with previous studies on rice^55^ and corn ^38^ that roots and leaves contained relatively high levels of THg.

**Table 3:** Total mercury (THg) and methylmercury (MeHg) content in soil and different tissues of *Houttuynia cordata* Thunb. (*HCT*) in Danzhai (DZ) and Zhijin (ZJ).

Items	Study area	Sample number	Root (DW, μg/kg)		Rhizome (DW, μg/kg)		Aboveground stem (DW, μg/kg)		Leaf (DW, μg/kg)		Soil (DW, μg/kg)	
			Range	AM ± SD	Range	AM ± SD	Range	AM ± SD	Range	AM ± SD	Range	AM ± SD
THg	DZ	14	172–1295	523 ± 357	53–219	109 ± 50	24–156	68 ± 34	169–659	260 ± 140	116–3440	756 ± 870
	ZJ	11	85–266	130 ± 59	15–76	29 ± 19	13–30	21 ± 6	25–61	40 ± 10	50–779	199 ± 203
MeHg	DZ	14	0.5–6.3	2.2 ± 1.8	0.4–3.4	1.6 ± 1.0	0.3–1.6	0. 7 ± 0.1	0.2–1.5	0.6 ± 0.4	0.9–7.3	2.8 ± 1.8
	ZJ	11	0.4–1.4	0.8 ± 0.3	0.3–0.7	0.4 ± 0.1	0.3–0.6	0.4 ± 0.1	0.1–0.6	0.3 ± 0.1	0.2–1.9	1.0 ± 0.5

*AM* arithmetic mean, *SD* standard deviation.

Interestingly, although roots had the highest THg content (the highest DW value being 1295 μg/kg), the average THg content in the underground parts was still lower than in the aboveground tissues. This is partly because the fibrous roots account for only 4.29 ± 1.3% (DW) of the total weight of underground parts, and partly because the leaves contain a relatively high THg content. Previous studies ^56,57^ have suggested that inorganic Hg in leaves is mainly derived from the atmosphere and this results also confirmed by other studies about paddy rice (*Oryza sativa* L.)^58^, corn (*Zea mays* L.) ^38^, and other plants ^56^, though some study found that plant leaf may also be a potential sources of atmospheric Hg^0^^59^. The average MeHg/THg ratio is 0.61 ± 0.45% for soil, which was comparable to the ratio in roots (0.52 ± 0.19%), and significantly higher (*p* < 0.05) than in leaves (0.41 ± 0.30%), and much lower than in the rhizomes (1.64 ± 0.69%) and the aboveground stems (1.52 ± 0.85%).

**Figure 4:**
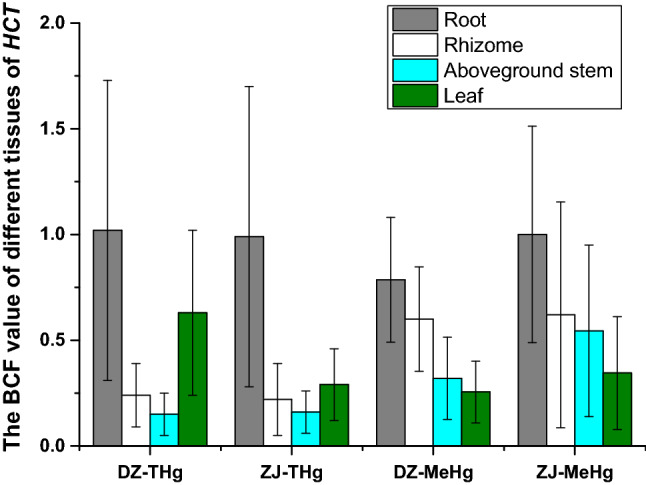
BCF value in different tissues of *Houttuynia cordata* Thunb. (*HCT*) in Danzhai(DZ) and Zhijin (ZJ).

The BCF value results are showed in Table S1 and Fig. 4. It could see that the highest BCF value for THg and MeHg in roots could reach 2.88 and 2.91, respectively (Table 3 and Fig. 4). The average THg BCF value in roots is 1.02 ± 0.71 in DZ and 0.99 ± 0.71 in ZJ, and that for MeHg is 0.79 ± 0.30 in DZ and 0.94 ± 0.51 in ZJ. Previous research by Qian et al. ^17^ shown that the highest BCF value of THg and MeHg among 259 wild plants could reached 5.5 and 18, respectively. Other research on rice ^60^ suggested that the BCF value of roots is 4.2 ± 2.1, 1.1 ± 0.8 for stems, and 0.72 ± 0.82 for leaves. The study of Zhang et al. ^58^ even showed that the BCF value for MeHg in rice could as much as 800 times higher than the value for inorganic Hg. This indicates that, for THg, *HCT* may be a high Hg accumulation plant, but its accumulation capacity for MeHg is relatively lower in comparison with other high MeHg accumulation plants. For both study areas of this study, the BCF value of THg followed the sequence of roots > leaves > rhizomes > aboveground stems, and MeHg followed the sequence of roots > rhizomes > aboveground stems > leaves. The sequence may confirm that part of inorganic Hg was transferred from soil through rhizomes to the aboveground stem and leaves and atmospheric Hg is another important Hg resources of foliage. It also suggested that soil MeHg was the only resources of MeHg in *HCT*.

Moreover, although *HCT* roots only account for a tiny share (4.29 ± 1.3%) of the weight of the underground parts, roots contributed 17.51 ± 7.83% (5.26–31.83%) and approximately 17.95 ± 3.47% (7.34–31.36%) of THg in the underground parts collected from DZ and ZJ, respectively. The equivalent values for MeHg were 5.82 ± 6.46% (3.4 2–10.10%) for DZ and 7.71 ± 3.47% (2.68–14.44%) for ZJ. Therefore, this level of potential Hg and MeHg exposure indicates that *HCT* roots should be removed before cooking.

### Correlations between Hg in soil and in different *HCT* tissues

Numerous studies have shown that soil is the major source of Hg in roots^38,55^ and the soil properties including pH value, organic matter, cation exchange capacity and soil Hg content may directly affected the Hg uptake by plants ^32,61^. The main soil types of this study are calcareous and yellow soil, which are enriched with aluminum and iron. SiO_2_, Al_2_O_3_ and Fe_2_O_3_ counted more than 80% of the soil profiles. We noted that the highest Hg content in *HCT* appeared at sampling sites 9, 10, and 11 (Fig. S5), which were located in the core area of the Danzhai Au-Hg mine, where the mining slag was a source of serious pollution in the local environment. The THg content in soil collected from DZ ranged from 159.5 to 3439.5 μg/kg (average, 755.8 ± 870.3 μg/kg), and only about 14% (N = 14) of samples breached the limit of 2 mg/kg above which contamination is considered unacceptable (Table 3)^62^. THg levels in soil samples collected from ZJ (N = 11) were all far below this limit (average, 204.0 ± 167.2 μg/kg), which is comparable to previous study about Hg content in soil where *HCT* is planted in Guiyang city (230 μg/kg)^63^.

Unitary linear regression analysis was used to analyze the correlation between Hg content in soil and different tissues in *HCT* (Table 4).Unlike previous studies of rice ^55^ and corn ^38^, which found that the main source of Hg in leaves is derived from the atmosphere and Hg transport from roots to leaves is limited ^64,65^, we found that the values of Hg content in different parts of *HCT* were all significantly correlated (*p* < 0.01) with soil Hg content. Significant correlations (*p* < 0.01) were obtained between THg and MeHg in the soil and roots (r^2^ = 0.615, *p* < 0.01; r^2^ = 0.836, *p* < 0.01), rhizomes (r^2^ = 0.499, *p* < 0.01; r^2^ = 0.816, *p* < 0.01), aboveground stems (r^2^ = 0.579, *p* < 0.01; r^2^ = 0.602, *p* < 0.01), and leaves (r^2^ = 0.498, *p* < 0.01; r^2^ = 0.414, *p* < 0.01). This indicated soil to be the major source of Hg in different *HCT* parts*,* including leaves, possibly because *HCT* has a creeping habit, thus facilitating the transport of Hg from the soil to leaves and this phenomenon is supported by the result in “THg and MeHg in the underground and aboveground parts of *HCT*” and “Hg accumulation in *HCT* tissues” and a previous study^23^ in artisanal and small-scale gold mining. Another study on Pb ^13^ in *HCT* found that the Pb content in leaves was much higher than in the stem and roots, although this element is nonvolatile, and this suggested that, as a creeping herbaceous plant, *HCT* has an efficient heavy metal transport capacity. Our results relating to underground parts (Table 4) are also in agreement with our previous study in Kaiyang county, Guizhou province ^12^, in which we found that Hg content in the main edible part (rhizome) of *HCT* was significantly correlated (r^2^ = 0.311, *p* < 0.01) with soil Hg content. The significant correlations between THg content in the soil and aboveground parts of *HCT* (r^2^ = 0.509, *p* < 0.01) should also be noted. Wang et al. ^66^ showed that the release of Hg from soil in an Hg mining area has led to a significantly higher total gas Hg concentration than other areas, and other studies^38,67^ confirmed that atmospheric Hg levels are an important resource for leaf Hg content of plants. Therefore, release of Hg from the soil is potentially an important source of Hg in leaves.

**Table 4:** Correlations between total mercury (THg) and methylmercury (MeHg) in soils and in different tissues of *Houttuynia cordata* Thunb. (*HCT*) in Danzhai (DZ) and Zhijin (ZJ).

Mercury species	Correlation analysis	Regress equation	Pearson coefficients
THg	Root	y = 0.375x + 1161.30	r^2^ = 0.615, p < 0.01, N = 25
	Rhizome	y = 0.057x + 44.27	r^2^ = 0.499, p < 0.01, N = 25
	Aboveground stem	y = 0.038x + 27.75	r^2^ = 0579, p < 0.01, N = 25
	Leaf	y = 0.169x + 96.80	r^2^ = 0.498, p < 0.01, N = 25
	Underground parts	y = 0.067x + 51.16	r^2^ = 0.490, p < 0.01, N = 25
	Aboveground parts	y = 0.140x + 81.82	r^2^ = 0.509, p < 0.01, N = 25
MeHg	Root	y = 0.828x -0.042	r^2^ = 0.836, p < 0.01, N = 25
	Rhizome	y = 0.508x + 0.064	r^2^ = 0.816, p < 0.01, N = 25
	Aboveground stem	y = 0.165x + 0.251	r^2^ = 0.602, p < 0.01, N = 25
	Leaf	y = 0.131x + 0.189	r^2^ = 0.414, p < 0.01, N = 25
	Underground parts	y = 1.07 × 10^-3^x + 0.49	r^2^ = 0.680, p < 0.01, N = 25
	Aboveground parts	y = 3.65 × 10^-4^x + 0.272	r^2^ = 0.695, p < 0.01, N = 25

### Risks of Hg exposure associated with *HCT* consumption

The parts of *HCT* considered edible differ among different areas of China: the aboveground parts are mainly consumed in Sichuan province and Chongqing city, while the underground parts are preferred in Guizhou, Yunnan, and Hunan provinces*.* Our results showed that *HCT* roots have the highest THg and MeHg contents (“Hg accumulation in *HCT* tissues”). Therefore, we assessed the Hg exposure risk from consuming *HCT* according to three scenarios: consuming the underground parts (CUP), the aboveground parts (CAP), or only the rhizomes (OCR). The ingestion rate (IR) value we choose was 76 g/person/day according to our previous study^12^.

**Figure 5:**
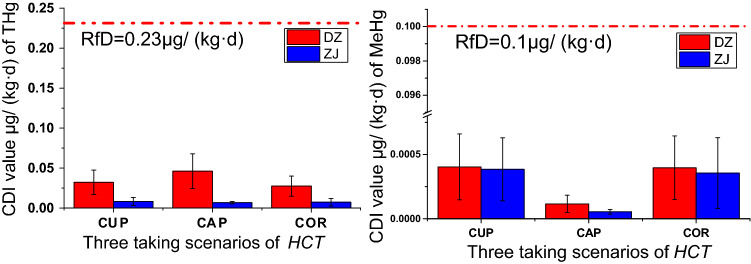
Chronic daily intake (CDI) values of (**a**) total mercury (THg) and (**b**) methylmercury (MeHg) under three *Houttuynia cordata* Thunb. (*HCT*) consumption scenarios.

CDI results clearly showed that substantial *HCT* consumption leads to relatively high health risks as a result of THg exposure (Fig. 5, Table S3); the highest CDI value reached over 1/3 of the reference dose (0.23 μg/kg/day) recommend by USEPA^68^.The exposure risk of THg in Hg mining area (DZ) was significant higher than that of non-Hg mining area (ZJ) (*p* < 0.05), while for MeHg, only the expose risk of CAP slight high than that of ZJ (Fig. 5). Both in Hg and non-Hg mining area, the exposure risk of THg of CAP was higher than of CUP, whereas the risk of exposure to MeHg from CAP was lower than that of CUP. The THg CDI values of the under- and aboveground parts accounts for 13.6% (range 7.6–29.2%) and 19.56% (range 10.9–42.9%), and for MeHg only 0.39% and 0.11% of the reference dose, respectively. While, this values of the under- and aboveground parts for THg accounted for only 3.6% and 2.9% and for MeHg accounted for 0.38% and 0.06% of the reference dose, respectively. This indicated a greater risk of THg exposure from *HCT* consumption in Hg mining areas, and a lower risk of MeHg exposure in both Hg and non-Hg mining areas.

Moreover, in Guizhou province, the rhizome is the main part of *HCT* consumed, but the root was not removed before cooking; this is not recommended since the root contains the highest levels of THg and MeHg. Table S2 and Fig. 4 showed the CDI values yielded by only consuming the rhizomes (OCR), the results indicated CDI values for THg are 14.3% (DZ) and 12.3% (ZJ), and for MeHg are 1.52% (DZ) and 23.1% (ZJ) less than that consuming the entire underground parts. The fibrous roots attached to the rhizome should therefore be removed before consumption.

## Conclusions

The risk of exposure to Hg by the consumption agricultural products is of great concern in Guizhou province because there are many Hg mining areas located. To our best knowledge, *HCT* is the only vegetable with high Hg- accumulation and been widely consumed in southwest China. Our results showed that THg contents in all parts and MeHg contents in underground parts of *HCT* in Hg mining area were significantly higher than that in the non-Hg mining area (control site). And the roots of *HCT* contained the highest THg and MeHg content. The BCF value of roots of *HCT* for THg and MeHg reached as high as 2.88 and 2.91, respectively. Hg contents in all tissues, including the leaves, were significantly correlated with soil Hg content, indicating that Hg pollution may have a major effect on the safety consumption of *HCT*. Consuming *HCT* from Hg mining area could be associated with a higher exposure risk to Hg and MeHg than that of non-Hg mining area. Preferred dietary habits in *HCT* consumption could directly affect the Hg exposure risk. Consuming the aboveground parts (CAP) of *HCT* potentially poses a high THg exposure risk and which is up to 40% of the reference dose in Hg mining areas. While, consuming the underground parts (CUP) may lead to a relatively high MeHg exposure risk both in Hg and no-Hg mining areas. All of our results indicate that this Hg accumulation plant should not be cultivated in Hg-contaminated areas, such as areas close to Hg slag or tailings, and another important factor is which part of *HCT* to be consumed, and it is recommended to remove the roots before cooking to reduce the Hg exposure risk.

## Supplementary Information

Supplementary information
