# Supplementary material for: Mercury accumulation in vegetable Houttuynia cordata Thunb. from two different geological areas in southwest China and implications for human consumption
Source: Sci Rep. 2021 Jan 8;11:52. doi: 10.1038/s41598-020-80183-7 (PMC7794452; doi:10.1038/s41598-020-80183-7)
Supplement: Supplementary file 1 — Supplementary information [file 41598_2020_80183_MOESM1_ESM.docx]

**Supporting information**

**Mercury accumulation in vegetable Houttuynia cordata Thunb. from two different geological areas in southwest China and implications for human consumption**

Qingfeng Wang^1,2*^, Zhonggen Li ^1,2^, Xinbin Feng^2,3^, Ao Wang^4^, Xinyu Li^2,3^, Dan Wang^1^, Leilei Fan^1^

1 Department of Resources and Environment, Zunyi Normal College, Zunyi 563006, P.R. China

2 State Key Laboratory of Environmental Geochemistry, Institute of Geochemistry, Chinese Academy of Sciences, Guiyang 550081, P. R. China

3 University of Chinese Academy of Sciences, Beijing 100049, P. R. China

4 Zunyi Product Quality Inspection & testing Institute, Zunyi 563000, P.R. China

-----------------------------------

*Corresponding author. Email address: qingfeng_424@163.com; Telephone: +86 0851-28950875; Fax: +86 0851-28950875; Address: Middle section of Pingan avenue, Xinpu town, Honghuagang district, Zunyi 563006, China


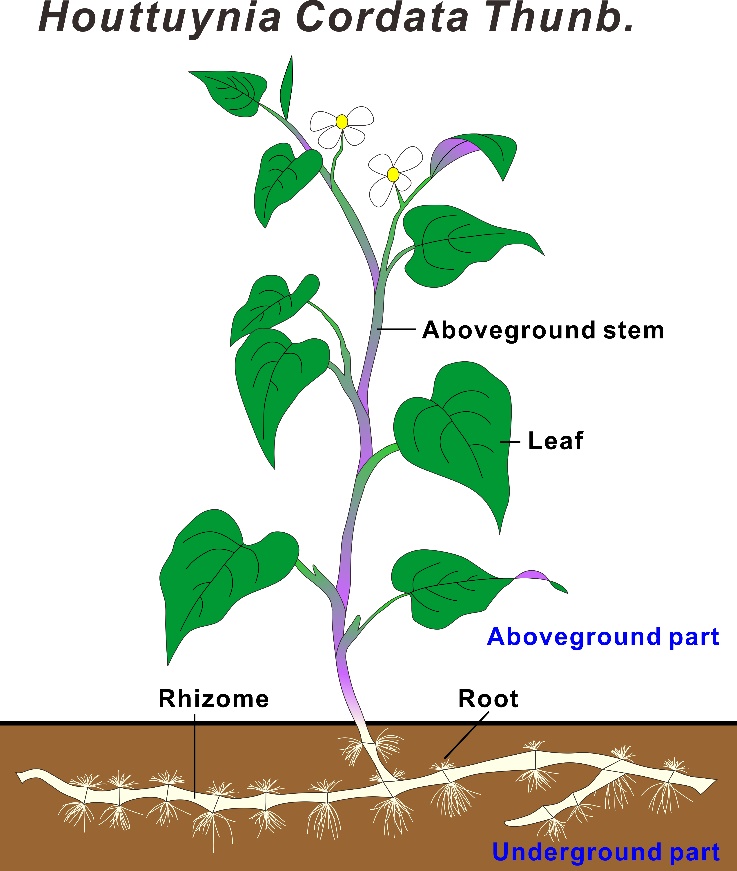


Fig.S1 the schematic diagram of each part of *HCT*


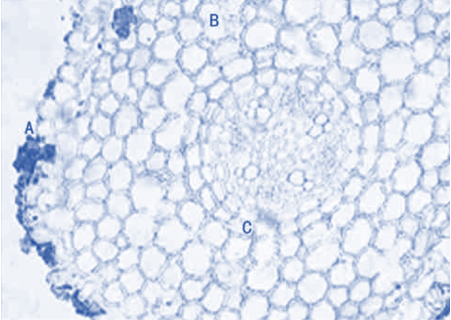


a (Yuan et al., 2002)

A. skin; B. Cortex; C. Endodermis


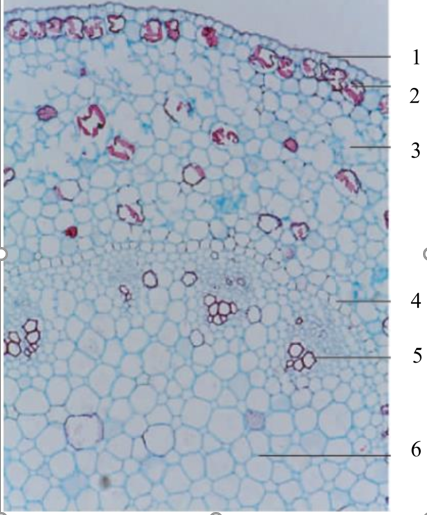


b (Lin et al., 2013)

1.Skin 2. Oil cell layer 3.Layer 4.Endodermis 5.Vascular bundle 6.pulp


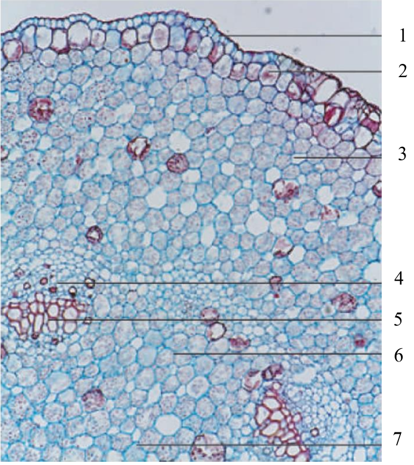


c. (Lin et al., 2013)

1.Skin 2.Oil cell layer 3.Layer 4.Phloem 5.Xylem 6.Medullary rays 7.pulp


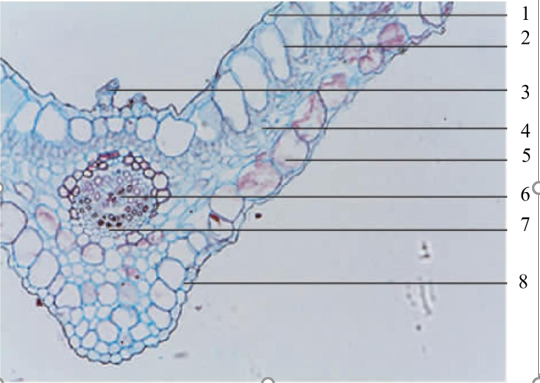


d (Lin et al., 2013)

1.Upper epidermis 2.Fence tissue 3.Non-glandular hairs 4.Spongy tissue 5.Oil cells 6.Xylem 7.Phloem 8.Under the skin

Fig.S2 Transverse of different tissues of *HCT*(10×10) a. root b. rhizome c. stem d. leaf

Fig.S3 Mercury concentration in underground and aboveground part of *HCT* among different sampling sites

a.THg(DW);b.MeHg(DW); c.THg, (FW); d.MeHg (FW).

Fig.S4 Mercury concentration in different tissues of *HCT* among different sampling sites

a.THg(DW);b.MeHg(DW); c.THg, (FW); d.MeHg (FW).

Fig.S5 THg(a) and MeHg(b) content in soil of different sampling sites

Table S1 The BFA value of THg and MeHg of different tissues of *HCT* in Danzhai and Zhiji

| Hg  forms | Aera | BAF value | | | |
| --- | --- | --- | --- | --- | --- |
|  |  | Root | Rhizome | Aboveground stem | Leaf |
| THg | DZ  N=14 | 1.02 ± 0.71  (0.31-2.88) | 0.24 ± 0.15  (0.06-0.58) | 0.15±0.10  (0.03-037) | 0.63 ± 0.39  (0.16-1.39) |
| MeHg |  | 0.79 ± 0.30  (0.35-1.35) | 0.60± 0.25  (0.30-1.12) | 0.32 ± 0.20  (0.08-0.83) | 0.26± 0.15  (0.10-0.49) |
| THg | ZJ  N=11 | 0.99 ± 0.71  (0.15 - 2.26) | 0.22 ± 0.17  (0.02 - 0.52) | 0.16 ± 0.10  (0.02 - 0.33) | 0.29 ± 0.17  0.05 - 0.55 |
| MeHg |  | 1.0 ± 0.51  (0.57-2.91) | 0.62 ± 0.53  (0.18-2.16) | 0.54 ± 0.41  (0.15-1.62) | 0.35 ± 0.27  (0.16-1.11) |

Table S2 the CDI value (μg/ (kg·d)) under different scenarios: eating underground parts (EUP), taking aboverground parts (TAP)and only consuming rhizome (OCR)

| Area | CDI value  μg/ (kg·d) | CUP | | CAP | | OCR | | Reference dose  (RfD)  (USEAP,1989) |
| --- | --- | --- | --- | --- | --- | --- | --- | --- |
|  |  | Range | AM±SD | Range | AM±SD | Range | AM±SD |  |
| DZ | THg | 0.018-0.069 | 0.032±0.015 | 0.026-0.101 | 0.046±0.022 | 0.013-0.055 | 0.028±0.013 | 0. 23 μg/ (kg·d) |
|  | MeHg | 1.13×10^-4^ -8.82×10^-4^ | 4.04×10^-4^ ± 2.56×10^-4^ | 0.57×10^-4^-2.61×10^-4^ | 1.16×10^-4^± 0.67×10^-4^ | 1.12×10^-4^-8.66×10^-4^ | 3.97×10^-4^± 2.48×10^-4^ | 0.1μg/(kg·d) |
| ZJ | THg | 0.005-0.021 | 0.008±0.005 | 0.005-0.010 | 0.007±0.0017 | 0.004-0.019 | 0.007±0.005 | 0. 23 μg/ (kg·d) |
|  | MeHg | 1.18×10^-4^-8.69×10^-4^ | 3.85×10^-4^ ± 2.45×10^-4^ | 0.24×10^-4^-0.95×10^-4^ | 0.55×10^-4^±0.19×10^-4^ | 1.01×10^-4^-7.25×10^-4^ | 3.56×10^-4^ ±0.26×10^-4^ | 0.1μg/(kg·d) |

AM: Arithmetic mean; SD: Standard deviation.

Table S3 Elemental characteristics of typical soils of the two area

| Sample | SiO_2_ | Al_2_O_3_ | Fe_2_O_3_ | TiO_2_ | K_2_O | Na_2_O | CaO | MgO | others |
| --- | --- | --- | --- | --- | --- | --- | --- | --- | --- |
| Danzai | 46.31 | 12.56 | 24.38 | 5.24 | 1.69 | 1.05 | 1.54 | 2.13 | 8.77 |
| Zhijin | 36.91 | 27.63 | 15.54 | 2.35 | 1.98 | 0.13 | 0.56 | 1.28 | 13.62 |
